# Immunohistochemistry is highly sensitive and specific for detection of *BRAF* V600E mutation in pleomorphic xanthoastrocytoma

**DOI:** 10.1186/2051-5960-1-20

**Published:** 2013-05-30

**Authors:** Cristiane M Ida, Julie A Vrana, Fausto J Rodriguez, Mark E Jentoft, Alissa A Caron, Sarah M Jenkins, Caterina Giannini

**Affiliations:** 1 Departments of Laboratory Medicine and Pathology, Mayo Clinic, Rochester, MN, USA; 2 Experimental Pathology, Mayo Clinic, Rochester, MN, USA; 3 Biomedical Statistics and Informatics, Mayo Clinic, Rochester, MN, USA; 4 Pathology, Johns Hopkins University, Baltimore, MD, USA

**Keywords:** Pleomorphic xanthoastrocytoma, *BRAF* V600E, Immunohistochemistry

## Abstract

**Background:**

High frequencies of the BRAF V600E mutation have been reported in pleomorphic xanthoastrocytoma (PXA). Recently, a BRAF V600E mutation-specific antibody has been developed and validated. We evaluated the immunohistochemical (IHC) detection of BRAF V600E mutation in PXA by comparing to gold standard molecular analysis and investigating the interobserver variability of the IHC scoring. We performed BRAF V600E IHC in 46 cases, of which 37 (80%) cases had sufficient tumor tissue for molecular analysis. IHC detection was performed using monoclonal mouse antibody VE1 (Spring Bioscience). IHC slides were scored independently by four reviewers blind to molecular data, including a primary (gold standard) and three additional reviewers. BRAF V600E mutation status was assessed by allele-specific polymerase chain reaction (PCR) with fragment analysis.

**Results:**

All 46 cases showed interpretable *BRAF* V600E IHC results: 27 (59%) were positive (strong cytoplasmic staining), 19 (41%) were negative (6 of these cases with focal/diffuse weak cytoplasmic staining, interpreted as nonspecific by the primary reviewer). By molecular analysis, all 37 cases that could be tested had evaluable results: 22 (59%) cases were positive for *BRAF* V600E mutation and were scored as “IHC-positive”, and 15 (41%) were negative (including 11 cases scored as “IHC-negative” and 4 cases scored as negative with minimal nonspecific staining). IHC detection of *BRAF* V600E mutant protein was congruent in all 37 cases that were successfully evaluated by molecular testing (sensitivity and specificity of 100%). Agreement for IHC scoring among the 4 reviewers was almost perfect (kappa 0.92) when cases were scored as “positive/negative” and substantial (kappa 0.78) when minimal nonspecific staining was taken into account.

**Conclusions:**

We conclude that detection of BRAF V600E mutation by immunohistochemistry is highly sensitive and specific. BRAF V600E IHC interpretation is usually straightforward, but awareness of possible nonspecific staining is necessary and training is recommended. It is a practical rapid method that may avoid the need of labor-intensive molecular testing and may be most valuable in small biopsies unsuitable for molecular analysis.

## Background

BRAF, a member of the RAF family including ARAF, BRAF and RAF1, is a serine/threonine protein kinase encoded by *BRAF* gene on chromosome 7q34 that activates the MAP kinase/ERK-signaling pathway mediating cellular responses to growth signals. It is the family member that is most easily activated by RAS, and the one with highest kinase activity [1-3]. Frequent somatic mutational activation of *BRAF* has been observed in human cancers, including melanomas, gliomas, colorectal cancers, lung cancers and others [4]. Among primary central nervous system (CNS) neoplasms [5-9], activation of the MAP kinase/ERK-signaling pathway appears to play an important role in the pathogenesis of a subset of glial/glioneuronal tumors, in particular, pilocytic astrocytoma (PA) [8-13], PXA [14], ganglioglioma (GG) [9,15], and dysembryoplastic neuroepithelial tumor (DNET) [16]. *BRAF* activation in PA primarily results from tandem duplications at 7q34 with subsequent fusion between the 5’ end of a gene of unknown function, *KIAA1549*, and the 3’ end of *BRAF*; while in PXA, GG and DNET, constitutive *BRAF* activation results from heterozygous missense mutation at codon 600 (V600E). *BRAF* V600E mutation is characterized by exchange of T to A at base position c.1799 (c. 1799 T > A), which results in substitution of glutamic acid by valine at residue 600 (p. Val600Glut). Less frequent activating *BRAF* mutations (e.g. V600K, V600D, V600M) have been observed in malignant melanoma [17-20] and other non-CNS tumors [4,21] but only rarely have been identified in primary CNS tumors [6].

The highest frequencies of *BRAF* V600E mutation in primary CNS neoplasms have been reported in PXA (up to 60-65%) [8,9,14,22], a WHO grade II tumor [23], with 30% recurrence and 80% overall survival rates at five years following primary resection. Histologically, PXA is characterized by marked cellular pleomorphism, nuclear atypia, and a variable number of bizarre, multinucleate giant cells (“classic PXA”), and occasionally shows increased mitotic activity and/or necrosis (“PXA with anaplastic features”) [23,24]. The main morphological differential diagnosis of PXA includes other pleomorphic and often more aggressive tumors such as glioblastoma (GBM)/giant cell or epithelioid GBM, a World Health Organization (WHO) grade IV tumor [23]. Such critical clinical distinction with important prognostic and clinical implications may be morphologically challenging. Of note, *BRAF* V600E mutation has been found in low frequency among GBM/giant cell GBM (approximately 5-10%) [5,7,14], but in up to 54% among epithelioid GBM [25]. Therefore, *BRAF* V600E mutation assessment may be a potentially useful marker in the differential diagnosis of GBM/giant cell GBM *vs*. “PXA with anaplastic features” and in identifying *BRAF* V600E mutant astrocytic tumors suitable for targeted therapy.

Recently, a *BRAF* V600E mutation-specific monoclonal antibody has been developed [26] and validated as a reliable test among tumors that frequently harbor the *BRAF* V600E mutation, including primary and metastatic melanoma [18,20,27,28], papillary thyroid carcinoma [29,30], hairy cell leukemia [31], ovarian serous borderline tumors [32], primary lung adenocarcinomas [33], as well as in a large series of brain metastases and corresponding non-CNS primary tumors [34].

Herein, we evaluated the IHC detection of BRAF V600E mutant protein in PXA by comparing to *BRAF* V600E mutation detection by molecular analysis, and investigated the interobserver variability of the IHC scoring. Detection of *BRAF* V600E mutation in PXA by immunohistochemistry was highly sensitive and specific, and showed a substantial/almost perfect interobserver agreement.

## Results

### *BRAF* V600E mutation immunohistochemical and molecular analyses

**Figure 1:**
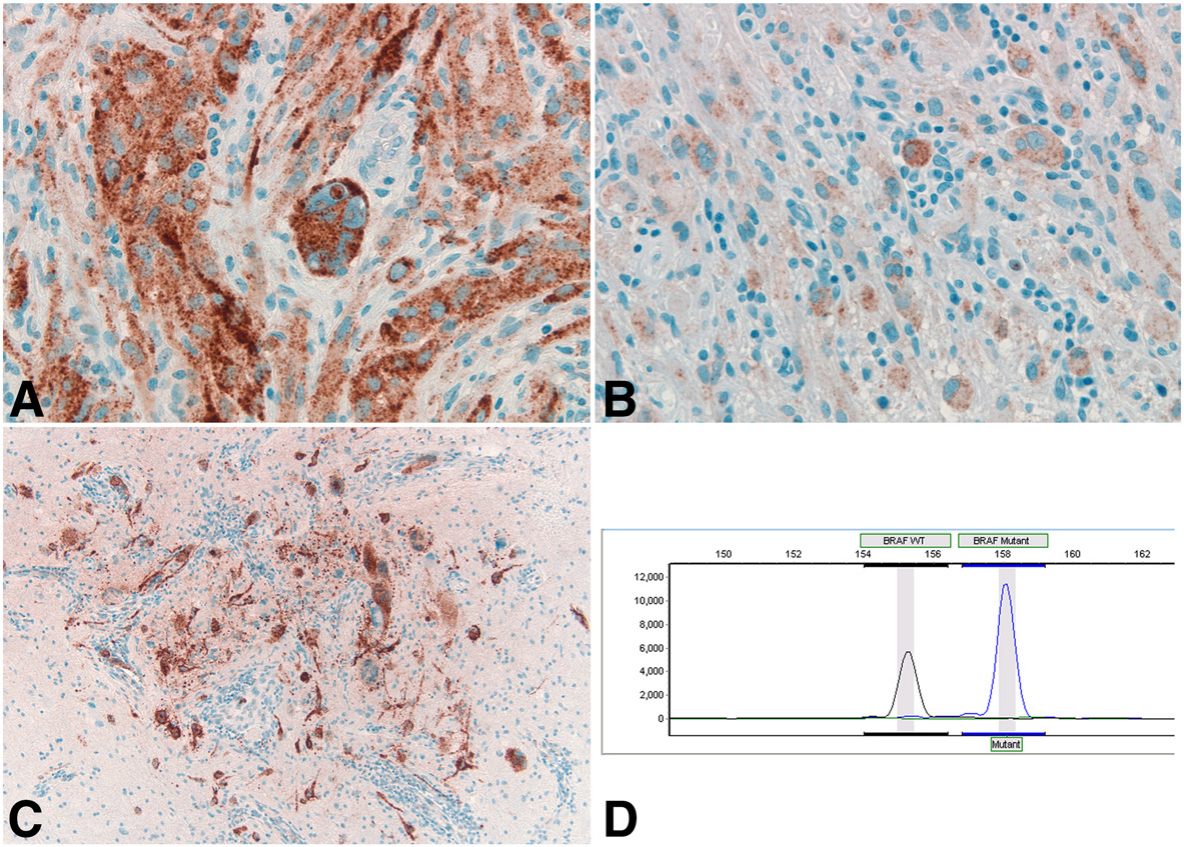
**BRAF V600E IHC positive PXA cases molecularly confirmed as *BRAF *** **V600E mutant tumors: *case 17*- strong granular cytoplasmic immunostaining of a characteristic pleomorphic multinucleated giant tumor cell (A), *case 3*- weak granular cytoplasmic immunostaining of pleomorphic and spindle tumor cells (B), *case 10*- strong granular cytoplasmic immunostaining of a cluster of isolated tumor cells (C), 400X; and *case 14*- mutant *BRAF* V600E peak in addition to the wild-type *BRAF* peak, consistent with presence of *BRAF* V600E mutation (D).**

**Figure 2:**
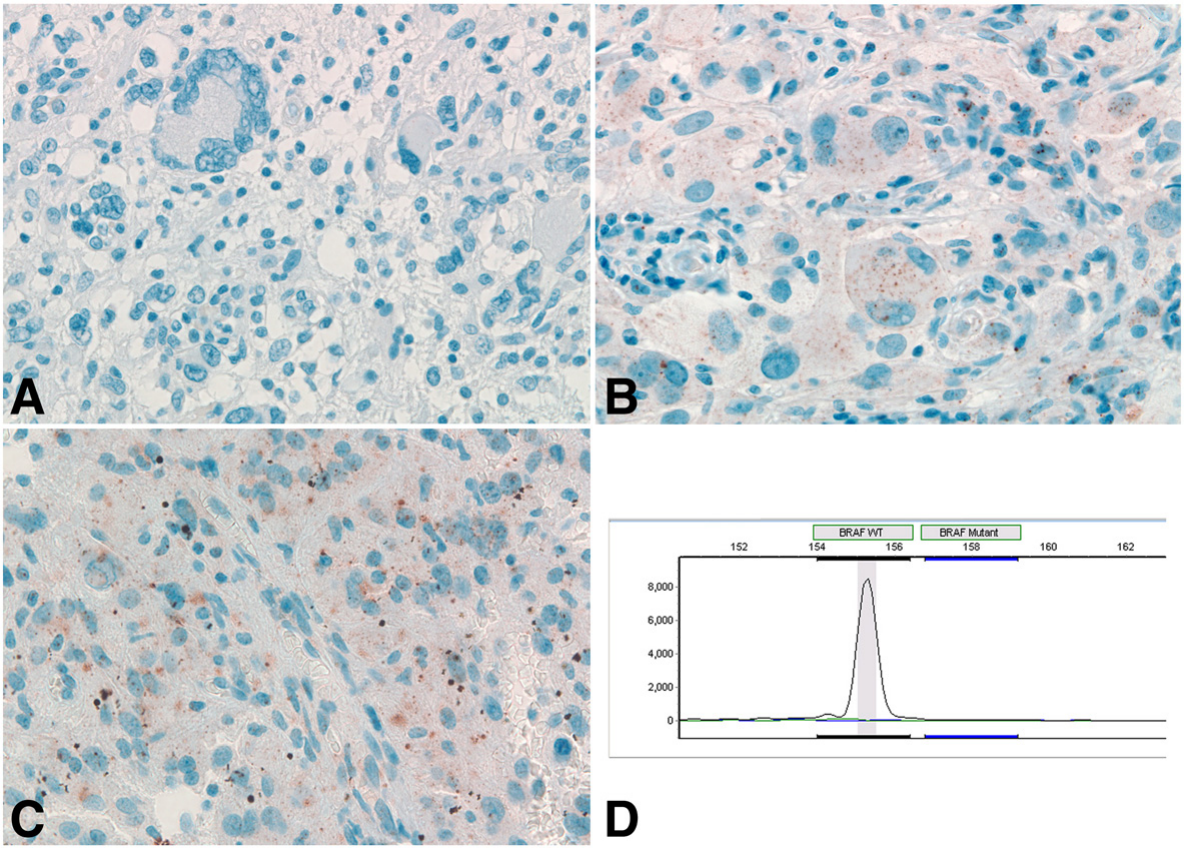
**BRAF V600E IHC negative & “negative/non-specific” PXA cases molecularly confirmed as *BRAF *** **V600E non-mutant tumors: *case 38*- BRAF V600E IHC negative case characterized by complete lack of tumor cell immunostaining (A), *case 20*- BRAF V600E IHC “negative/non-specific” case showing focal weak granular cytoplasmic immunostaining of tumor cells (B), *case 47*- BRAF V600E IHC “negative/non-specific” case with focal weak granular cytoplasmic immunostaining of tumor cells in addition to small extracellular immunostaining precipitate background (C); 400X; and *case 20*- *BRAF* wild-type peak without mutant *BRAF* V600E peak, consistent with the absence of *BRAF* V600E mutation and in keeping with the negative BRAFV600E IHC (D).**

All 46 (100%) cases with available tissue were evaluable by immunohistochemistry for BRAF V600E mutant protein. Cases were scored as positive if non-ambiguous tumor cell cytoplasmic staining was identified (“IHC-positive”). BRAF V600E immunoreactivity in most cases was characterized by intense cytoplasmic stain, with a somewhat granular appearance, likely due to the visualization/amplification set used and with variable distribution. In some cases, all tumor cells stained; while in others, the stain was patchy in the tissue sections. Isolated tumor cells infiltrating parenchyma were also visualized by the immunostain. According to the primary reviewer, 27 (59%) cases were “IHC-positive,” and 19 (41%) cases were “IHC-negative,” including 6 cases “IHC-negative/nonspecific.” Examples of immunostaining pattern are shown in Figures 1 and 2. Immunostaining varied in intensity from strong (Figure 1A) to weak (Figure 1B), as well as in distribution, with cases showing isolated infiltrating tumor cells (Figure 1C). Among cases considered negative (“IHC-negative”), although the majority of cases did not have any degree of immunostaining (Figure 2A), a few showed very focal and/or weak tumor cell cytoplasmic staining as shown in Figures 2B and 2C. This focal/weak immunostaining was interpreted as nonspecific (“IHC-negative/nonspecific”).

Among the 37 (of 46, 80%) cases with sufficient tumor tissue for molecular analysis: 22 (of 37, 59%) were positive for *BRAF* V600E mutation, including 16 classic PXA and 6 PXA with anaplastic features, and 15 (41%) cases were negative for *BRAF* V600E mutation, including 7 classic PXA and 8 PXA with anaplastic features. All 22 cases positive for *BRAF* V600E mutation (Figure 1D; Additional file 1: Figure S1 for complete electropherogram panel) had been scored as “IHC-positive.” Of the 15 cases negative for *BRAF* V600E mutation (Figure 2D; Additional file 2: Figure S2 for complete electropherogram panel), 11 had been scored as “IHC-negative” and 4 as “IHC-negative/nonspecific” cases (Table 1). Therefore, there was complete agreement between *BRAF* V600E mutation molecular analysis and IHC detection of BRAF V600E mutant protein in all 37 cases, with sensitivity of 100% (confidence interval 85.1-100) and specificity of 100% (79.6-100) according to the primary reviewer.

**Table 1:** ***BRAF *** **V600E mutation and BRAF V600E IHC (Primary reviewer) comparison (n = 37)**

***BRAF *** **V600E Mutation**	**BRAF V600E IHC**		
	**Positive**	**Negative**	**Negative/Nonspecific**
Mutant	22	0	0
Non-mutant	0	11	4

### Interobserver variability of BRAF V600E immunohistochemical analysis

**Figure 3: Table 2:**
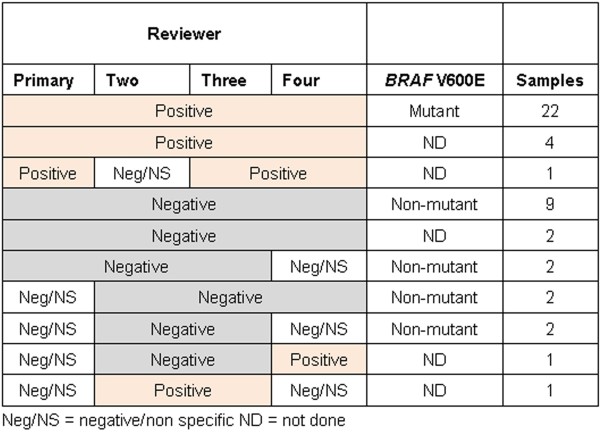
**BRAF V600E IHC Interobserver variability (n = 46).** Neg/NS = negative/non specific. ND = not done.

Most (26) cases scored as “IHC-positive” by the primary reviewer were also interpreted as “IHC-positive” by the other three reviewers (Figure 3: Table 2 ), with the exception of one “IHC negative/nonspecific” interpretation by a single reviewer. However, reviewers three and four also scored as positive one additional case among those scored as “IHC-negative/nonspecific” by the primary reviewer (Figure 3: Table 2 ). All 13 cases scored as “IHC-negative” by the primary reviewer were also scored as “IHC-negative” by reviewers two and three, while reviewer four scored two of them as “IHC-negative/nonspecific.” The results of the review of the remaining six cases interpreted as “IHC-negative/nonspecific” by the primary reviewer as well as the results of the *BRAF* V600E mutation molecular analysis are detailed in Figure 3: Table 2. In summary: two cases were scored as “IHC-negative” by the other three observers; two cases were also scored as “IHC-negative/nonspecific” by one observer and as “IHC-negative” by the other two observers; one case was scored as “IHC-negative” by two observers and as “IHC-positive” by one observer; one case was also scored as “IHC-negative/nonspecific” by one observer and as “IHC-positive” by the other two observers.

Agreement for IHC scoring was almost perfect (kappa 0.92) when cases were scored as “positive/negative” (“IHC-positive” *vs*. “IHC-negative”) and substantial (kappa 0.78) when “negative/nonspecific” staining was taken into account (“IHC-positive” *vs*. “IHC-negative” *vs*. “IHC-negative-nonspecific”).

## Discussion

In keeping with the majority of previous studies that also evaluated the immunohistochemical detection of the *BRAF* V600E mutation in comparison to gold standard molecular testing in a variety of tumor types [16,18,20,26-34], we concluded that immunohistochemistry is an accurate detection method for the *BRAF* V600E mutation in PXA. In our study, IHC results were comparable to the gold standard molecular testing for identification of *BRAF* V600E mutation and resulted in high sensitivity and specificity. In contrast to the labor-intensive detection of *BRAF* V600E mutation by molecular testing, identification of BRAF V600E mutant protein by immunohistochemistry is a rapid method that may be quickly implemented in diagnostic pathology practice since it is a widespread technique available in most academic centers as well as in non-academic pathology practices. However, validation of the BRAF V600E mutation IHC specificity by comparative molecular analysis should be performed for each tumor entity before routine diagnostic implementation, since it has been recently reported that in pituitary adenomas, the *BRAF* V600E mutation-specific VE1 immunostaining is not associated with presence of *BRAF* V600E mutation [35].

In surgical neuropathology, *BRAF* V600E mutation-specific immunohistochemistry has a potential clinically relevant role. IHC detection of BRAF V600E mutant protein is an accurate and reliable alternative method that may be diagnostically useful when dealing with morphologically challenging pleomorphic astrocytic tumors in which the differential diagnoses include PXA and GBM/giant cell GBM. In addition, *BRAF* V600E mutation has also been identified to a lesser extent in epithelioid GBM[25], ganglioglioma and pilocytic astrocytoma, predominantly in extra-cerebellar location [9], expanding the diagnostic repertoire use of BRAF V600E mutation-specific IHC testing among primary CNS tumors and potentially the identification of tumors suitable for *BRAF* V600E mutation-targeted therapy.

Of note, low tumor cell content may prevent identification of the *BRAF* V600E mutation by molecular analysis and result in false-negative results, but this limitation has been shown to be overcome by the use of BRAF V600E monoclonal antibody [18,29,30,32,34]. Thus, small biopsies that would be deemed unsuitable for molecular testing may be successfully evaluated by immunohistochemistry. This is particularly important in surgical neuropathology, in which biopsy samples of small size and/or with low tumor cell content are not infrequent. In addition, BRAF V600E IHC analysis allows identification of tumor cells at the single cell level, which would not only aid in the diagnosis of biopsies with very low tumor cell content but would also allow localization of the mutation at a cellular type level, as recently highlighted by the demonstration of neuronal tumor cells as the predominant tumor cell population harboring the *BRAF* V600E mutation in gangliogliomas [15].

In our study, BRAF V600E IHC interpretation was usually straightforward with high (at least substantial) interobserver agreement. The presence of rare cases with nonspecific staining is a potential pitfall, which could lead to rare false positive results. Similarly, others have also reported negative cases with nonspecific background staining [15,32,34]. In ambiguous cases, sequential molecular confirmatory testing is advocated [20,27]. We recommend that pathologists not only be aware of the possibility of nonspecific staining but also train during validation of the IHC testing to correctly identify such cases and avoid misinterpretation of nonspecific staining as positive staining.

## Conclusions

Detection of *BRAF* V600E mutation in PXA by immunohistochemistry is highly sensitive and specific with substantial/almost perfect interobserver agreement. BRAF V600E IHC interpretation is usually straightforward, but awareness of possible nonspecific staining is necessary, and training on the IHC stain interpretation is recommended. It is a practical rapid method that may avoid the need of labor-intensive molecular testing and may be extremely valuable in biopsies unsuitable for molecular analysis due to either small size or low tumor cell content.

## Methods

### Case selection

All studies were conducted according to Mayo Institutional Review Board–approved protocols. This study was approved by the Mayo IRB as a minimal risk study with waiver of consent.

According to the Minnesota law, the Minnesota research authorization status was reviewed and only patients whose Research Status was "yes" were included in the study.

Fifty pleomorphic xanthoastrocytoma (PXA) cases upon first surgical resection were selected from the files from Mayo Clinic (n = 32) and Johns Hopkins University (n = 18) from 1965 to 2011. All existing diagnostic slides were retrieved and reviewed by at least two of the authors (C.G. and C.M.I.), and the diagnosis of PXA was confirmed according to previously described criteria [23]. Tumors showed a relatively solid growth pattern and were composed of a combination of spindle-shaped, xanthic and pleomorphic, multinucleated giant astrocytes, associated with both pale and bright eosinophilic granular bodies. They included both classic PXA (≤5 mitotic figures per 10 high power fields) and PXA with anaplastic features (including >5 mitotic figures per 10 high power fields and/or necrosis). All 50 cases were classified as either classic PXA (n = 34) or PXA with anaplastic features (n = 16) and were included in the study. Of these, 46 cases (92%) had available tissue for IHC and/or molecular analysis.

### BRAF V600E immunohistochemical analysis

Four-micron freshly cut sections (<2 weeks) of formalin-fixed, paraffin-embedded (FFPE) tissue of 46 (of 50) cases were dried and melting at 62°C oven for 20 minutes. Subsequently, they were stained with mouse monoclonal BRAF V600E antibody (1/100 titer; clone VE1) and raised against a synthetic peptide corresponding to amino acids 596–606 (GLATEKSRWSG) of mutant BRAF (Spring Bioscience) with slight modifications to the manufacturer’s protocol. Briefly, staining was performed on the Ventana BenchMark XT (Ventana Medical Systems Inc.). The staining protocol included online deparaffinization, HIER (Heat Induced Epitope Retrieval) with Ventana Cell Conditioning 1 for 32 minutes and primary antibody incubation for 32 minutes at 37°C. Antigen-antibody reactions were visualized using Ventana OptiViewTM Amplification kit, followed by Ventana OptiViewTM Universal DAB Detection Kit (Optiview HQ Linker 8 min, Optiview HRP Multimer 8 min, Optiview Amplifier H2O2/Amplifier 4 min, Optiview Amplifier Multimer 4 min, Optiview H2O2/DAB 8 min, Optiview Copper 4 min). Counterstaining was obtained online using Ventana Hematoxylin II for 8 minutes followed by bluing reagent for 4 minutes. Finally, all slides are removed from the stainer, dehydrated, and coverslipped for microscopic examination. Positive control included a known *BRAF* V600E mutant skin malignant melanoma. Cases were scored as positive (“IHC-positive”), negative (“IHC-negative”), and “negative-nonspecific” (“IHC negative/nonspecific”). Only tumor cells showing non-ambiguous cytoplasmic staining for BRAF V600E immunostain were scored as positive (“IHC positive”). Faint, weak granular stain was noted in a few cases and considered nonspecific. These cases were scored as “IHC negative/nonspecific” for tracking purposes. IHC slides were scored first by a primary reviewer and later by three additional independent reviewers, all four blind to the molecular results. The primary reviewer (CG) was the one most intimately involved with the development of the immunostain, who had gained most experience through the process, and was most familiar with variations in stain intensity and distribution not only among PXA cases but also with other positive and negative controls. Because of the level of expertise with reading the BRAF V600E immunostain and of the sequence of events, the primary reviewer was used as the “gold standard” to which the results of the *BRAF* V600E mutation molecular analysis were compared (see below). The four reviewers were compared to each other in regards to the scoring of BRAF V600E immunohistochemistry.

### BRAF V600E mutation status by allele-specific PCR with fragment analysis

After review of hematoxylin and eosin-stained slides, of all the 50 cases, 37 (74%) cases had sufficient (≥20%) viable tumor for *BRAF* V600E mutation molecular analysis. DNA was extracted from 5-micron sections of FFPE tissue (four to eight slides per case) using the QIAamp DNA FFPE Tissue Kit (Qiagen) according to the manufacturer's instructions with few modifications (samples were lysed overnight; 10 uL of additional proteinase K was used on the following day; the tissue was allowed to complete lysis for additional one to two hours). DNA was quantified using a Qubit fluorometer and Qubit Quant-iT dsDNA BR Assay Kit (Invitrogen). Testing for the BRAF V600E mutation was performed following clinically validated protocols. *BRAF* allele-specific fluorescent PCR was performed with primers specifically designed to detect the base mutant and wild type base at position c.1799, and fragment analysis was completed on the ABI 3730 DNA Analyzer (Applied Biosystems). The primers were differentially labeled and had a size variance in addition to the different fluorophore. Wild-type peak was approximately 155.2 base pairs, and the mutant peak was approximately 158 base pairs (predetermined bins were set at +/− 1.2 base pairs from the expected size, i.e. 155.2 +/−1.2 bp and 158 +/−1.2 bp).

### Statistical analysis

Sensitivity and specificity along with 95% confidence intervals (score method) were calculated for BRAF V600E IHC analysis based on the reading of the primary reviewer compared to the *BRAF* V600E mutation molecular analysis result considered the “gold standard”.

To evaluate the degree of agreement for IHC interpretation among the four observers, overall kappa for >2 reviewers were calculated. Kappa values may vary from 0 to 1.0. Values of 0.4 to 0.6 are considered evidence of “moderate” agreement, >0.6-0.8 of “substantial,” and >0.8-1 of “almost perfect” agreement according to Landis and Koch [36]. Kappa was calculated based on the IHC scoring results in two scenarios: when cases were scored as “positive/negative” and when negative/nonspecific staining was also taken into account [36-39]. All analyses were performed using SAS version 9 (Cary, NC).

## Abbreviations

PXA: Pleomorphic xanthoastrocytoma; IHC: Immunohistochemical; CNS: Central nervous system; PA: Pilocytic astrocytoma; GG: Ganglioglioma; DNET: Dysembryoplastic neuroepithelial tumor; WHO: World Health Organization; GBM: Glioblastoma; HIER: Heat-Induced Epitope Retrieval; PCR: Polymerase chain reaction; BP: Base pairs

## Competing interests

The authors declare that they have no competing interests.

## Authors’ contributions

Cristiane M. Ida, MD, planned project, reviewed cases, wrote manuscript. Julie A. Vrana performed BRAF V600E immunostaining, reviewed manuscript. Fausto J. Rodriguez, MD, provided and reviewed part of the cases, reviewed manuscript. Mark E. Jentoft, MD, reviewed immunohistochemical stains, reviewed manuscript. Alissa A. Caron performed DNA extraction for *BRAF* V600E mutation molecular analysis, reviewed manuscript. Sarah M. Jenkins performed statistical analysis, reviewed manuscript. Caterina Giannini, MD, planned project, reviewed cases, prepared figures, wrote manuscript. All authors read and approved the final manuscript.

## Supplementary Material

Additional file 1: Figure S1 Fragment analysis electropherograms of a *BRAF* V600E mutant case (case 14): mutant *BRAF* V600E peak in addition to the wild- type *BRAF* peak, consistent with presence of *BRAF* V600E mutation (**A**); *positive control*- mutant *BRAF* V600E peak in addition to the wild-type *BRAF* peak, consistent with presence of *BRAF* V600E mutation (**B**); *negative control*- *BRAF* wild-type peak without mutant *BRAF* V600E peak, consistent absence of *BRAF* V600E mutation (**C**); *Blank/No DNA-* Absence of distinct peaks, indicating no sample contamination (**D**). Click here for file

Additional file 2: Figure S2 Fragment analysis electropherograms of a *BRAF* V600E non-mutant case (case 20): *BRAF* wild-type peak without mutant *BRAF* V600E peak, consistent with absence of *BRAF* V600E mutation (**A**); *positive control*- mutant *BRAF* V600E peak in addition to the wild-type *BRAF* peak, consistent with presence of *BRAF* V600E mutation (**B**); *negative control*- *BRAF* wild-type peak without mutant *BRAF* V600E peak, consistent absence of *BRAF* V600E mutation (**C**); *Blank/No DNA-* Absence of distinct peaks, indicating no sample contamination (**D**). Click here for file
