# Supplementary figures and images for: Immunohistochemistry is highly sensitive and specific for detection of BRAF V600E mutation in pleomorphic xanthoastrocytoma
Source: Acta Neuropathol Commun. 2013 May 30;1:20. doi: 10.1186/2051-5960-1-20 (PMC3893490; doi:10.1186/2051-5960-1-20)

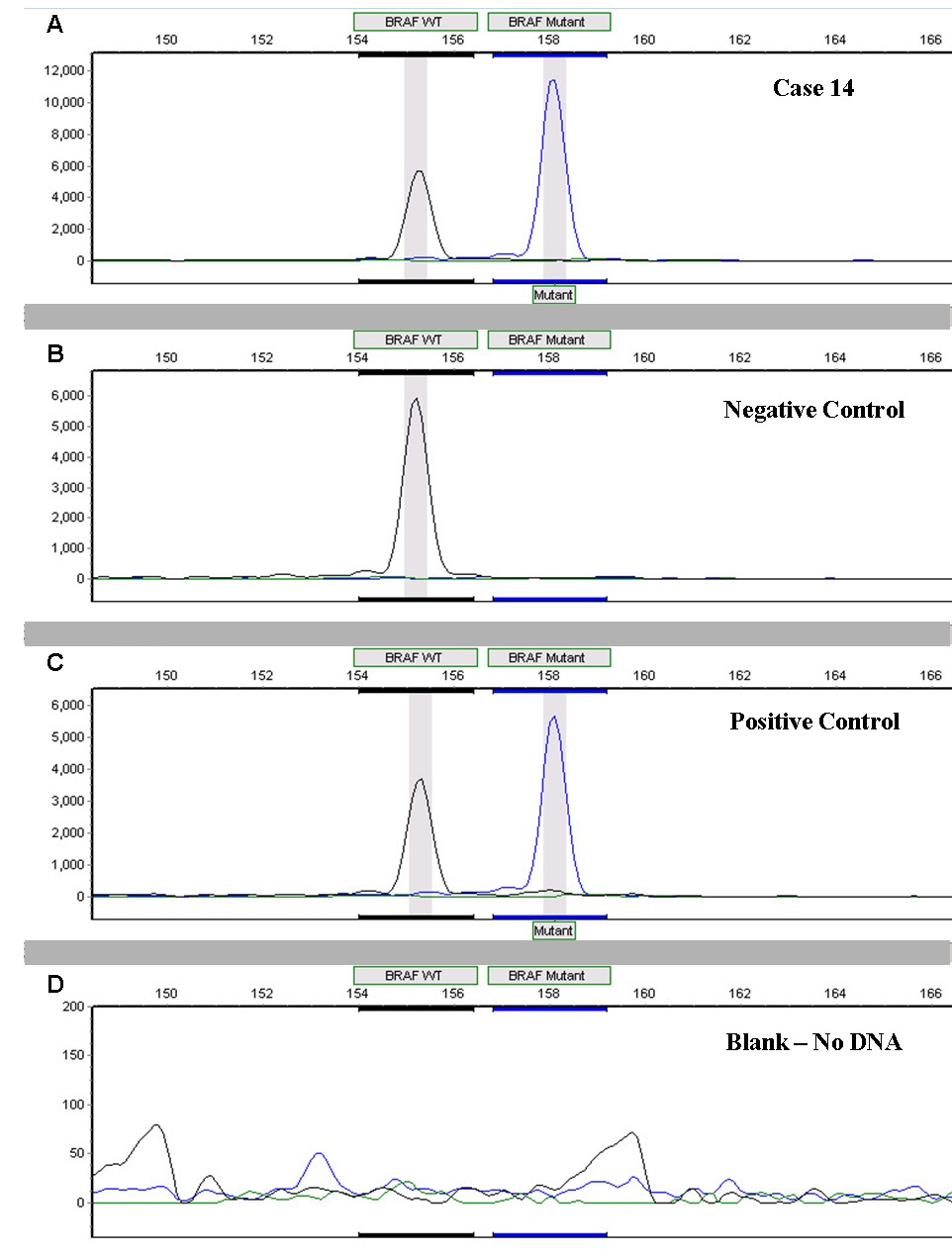

Supplement: Additional file 1: Figure S1 — Fragment analysis electropherograms of a BRAF V600E mutant case (case 14): mutant BRAF V600E peak in addition to the wild- type BRAF peak, consistent with presence of BRAF V600E mutation (A); positive control- mutant BRAF V600E peak in addition to the wild-type BRAF peak, consistent with presence of BRAF V600E mutation (B); negative control- BRAF wild-type peak without mutant BRAF V600E peak, consistent absence of BRAF V600E mutation (C); Blank/No DNA- Absence of distinct peaks, indicating no sample contamination (D). [file 2051-5960-1-20-S1.tiff]

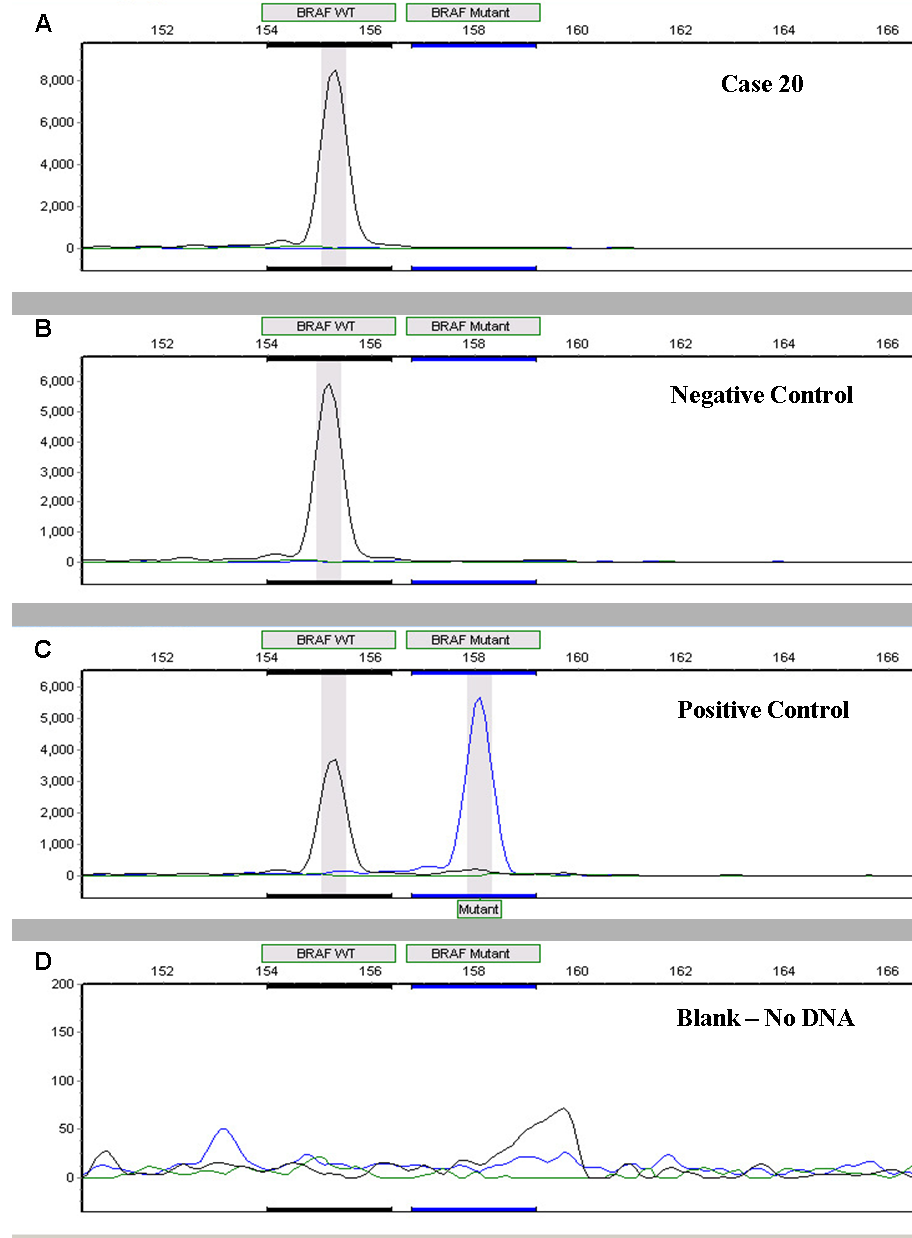

Supplement: Additional file 2: Figure S2 — Fragment analysis electropherograms of a BRAF V600E non-mutant case (case 20): BRAF wild-type peak without mutant BRAF V600E peak, consistent with absence of BRAF V600E mutation (A); positive control- mutant BRAF V600E peak in addition to the wild-type BRAF peak, consistent with presence of BRAF V600E mutation (B); negative control- BRAF wild-type peak without mutant BRAF V600E peak, consistent absence of BRAF V600E mutation (C); Blank/No DNA- Absence of distinct peaks, indicating no sample contamination (D). [file 2051-5960-1-20-S2.tiff]
